# Predictors of Disease Progression in Idiopathic Pulmonary Fibrosis Under Antifibrotic Therapy: A Retrospective Study

**DOI:** 10.7759/cureus.99813

**Published:** 2025-12-22

**Authors:** Tomoo Kishaba, Mariko Higa, Hiroaki Nagano

**Affiliations:** 1 Department of Respiratory Medicine, Okinawa Chubu Hospital, Uruma City, JPN; 2 Department of Internal Medicine, Ikigai Zaitaku Clinic, Okinawa City, JPN

**Keywords:** antifibrotic therapies, disease progression, ipf, peak expiratory flow, total lung capacity

## Abstract

Background: Idiopathic pulmonary fibrosis (IPF) is a progressive fibrosing lung disease with a low median survival of three to five years. Antifibrotic agents, pirfenidone and nintedanib, slow but do not reverse disease progression, and treatment response varies. Identifying baseline predictors of progression may help personalize care. Methods: We retrospectively analyzed consecutive IPF patients treated at Okinawa Chubu Hospital between 2012 and 2020. Eligible patients received antifibrotics for ≥3 months and had baseline and follow-up pulmonary function tests (PFTs). Clinical, laboratory, PFT, and high-resolution computed tomography (HRCT) data were collected. Disease progression within one year was defined as ≥2 of: worsening symptoms, ≥5% decline in forced vital capacity (FVC), or radiologic progression. Predictors were evaluated using exact logistic regression; survival was assessed with Kaplan-Meier analysis. Results: Forty-seven patients (mean age 73.3 years, 32 men) were included; 63.8% were ever-smokers. Mean baseline FVC was 1.93 L, %FVC 67.6%, % peak expiratory flow (PEF) 75.0%, %total leucocyte count (TLC) 72.4%, %diffusing capacity of the lungs for carbon monoxide (*DLCO*) 63.7%. HRCT showed definite usual interstitial pneumonia (UIP) in 20 patients, probable UIP in 19, and indeterminate UIP in eight. Progression occurred in 29 patients (61.7%). Lower baseline %PEF (OR 0.977, p=0.097) and %TLC (OR 0.953, p=0.071) were associated with early progression. Median survival was 47 months; patients with preserved %PEF and %TLC had a slower decline. Conclusions: Lower %PEF and %TLC may predict poorer response to antifibrotics in IPF. These readily available PFT indices could serve as practical markers for risk stratification and early intervention. Prospective, multicenter studies are warranted to confirm predictive value.

## Introduction

Idiopathic pulmonary fibrosis (IPF) is the most severe and fatal form of restrictive interstitial lung disease. Historically, treatments focused on anti-inflammatory agents such as corticosteroids, but these offered limited efficacy and carried significant risks [1]. A therapeutic breakthrough arrived with the introduction of antifibrotic agents: pirfenidone (approved in Japan in 2008) and nintedanib (approved in 2014), which were shown in landmark randomized controlled trials, ASCEND (Assessment of Pirfenidone to Confirm Efficacy and Safety in Idiopathic Pulmonary Fibrosis) and INPULSIS, to slow the decline in forced vital capacity (FVC) and improve progression-free survival [2,3]. These advances formed the basis for the 2018 American Thoracic Society (ATS), European Respiratory Society (ERS), Japanese Respiratory Society (JRS), and Latin American Thoracic Association (ALAT) guidelines, which endorse antifibrotic therapy as standard of care for IPF [4]. Notably, these guidelines also emphasize the need for robust prognostic markers to guide clinical management and identify patients at high risk of rapid progression.

Despite these advances, IPF remains a life-limiting disease, with a median survival of just three to five years [5,6]. Even with antifibrotic treatment, a subset of patients experiences rapid disease progression. This clinical behavior mirrors the phenotype of “progressive pulmonary fibrosis” (PPF), a term recognized in the updated 2022 ATS/ERS/JRS/ALAT guidelines to describe a progressive pattern of fibrosis across interstitial lung diseases beyond IPF [6].

The recognition of PPF underscores the urgency of identifying prognostic markers for rapid progression despite therapy, enabling timely intervention [7-9]. To address this unmet need, we conducted a retrospective analysis in antifibrotic-treated IPF patients. Objective: To identify baseline clinical, physiologic, and radiologic factors associated with disease progression within one year.

Although FVC and diffusing capacity of the lungs for carbon monoxide (DLCO) are most commonly used for prognostication in IPF, additional spirometric and lung-volume measures may capture complementary aspects of respiratory mechanics and functional reserve. Peak expiratory flow (%PEF) reflects the early expiratory portion of the flow-volume curve and is influenced by respiratory muscle performance, airway mechanics, and lung elastic recoil; emerging data suggest that reduced peak expiratory flow rate (PEFR) or atypical flow-volume curve morphology may be associated with adverse outcomes in IPF. Total lung capacity (%TLC) represents the global restrictive burden and may vary independently from FVC in some patients; lung-volume-based indices (including right ventricular (RV)/TLC as a marker of air trapping) have also been explored in IPF cohorts with respect to disease severity and outcomes. We therefore examined baseline %PEF and %TLC as readily available, potentially complementary physiologic markers for identifying patients at higher risk of early progression despite antifibrotic therapy.

## Materials and methods

We conducted a retrospective observational study at Okinawa Chubu Hospital, a tertiary referral center in Okinawa, Japan. The study population consisted of consecutive patients who were clinically and radiologically diagnosed with IPF according to the 2011 ATS/ERS/JRS/ALAT consensus criteria [4,10] and were treated between January 2012 and December 2020. Chronic obstructive lung disease was excluded using pulmonary function tests (PFTs). Therefore, the whole cohort revealed FEV in one second (FEV1) of 70%. The study was approved by the Okinawa Chubu Hospital Institutional Review Board. Informed consent was waived because of the retrospective study.

Study population

To be eligible for inclusion, patients had to have been initiated on antifibrotic therapy, either nintedanib or pirfenidone, and continued treatment for a minimum of three months. Patients also needed to have completed PFTs at both baseline (prior to or shortly after treatment initiation) and at follow-up, allowing for assessment of disease trajectory. Patients with incomplete clinical data or follow-up loss within one year were excluded. Antifibrotic agent selection (nintedanib or pirfenidone) and dosing were determined by treating physicians as part of routine clinical practice; detailed longitudinal treatment exposure (dose intensity, dose reductions, temporary interruptions, and discontinuation) was not systematically available for analysis. Patients lost to follow-up within one year were excluded.

Data collection

Comprehensive baseline data were collected, including demographic characteristics, smoking history, comorbidities, and physical symptoms. Laboratory parameters such as white blood cell (WBC) count, serum lactate dehydrogenase (LDH), and Krebs von den Lungen-6 (KL-6) levels were measured [11,12]. PFT indices included FVC, %FVC, %PEF, percent predicted functional residual capacity (%FRC), %TLC, and %DLCO. Additionally, two composite physiologic scoring systems were calculated: the Gender-Age-Physiology (GAP) index and the Composite Physiologic Index (CPI), both of which are validated tools for prognostication in IPF [5,13-16].

Data analysis and definitions

High-resolution computed tomography (HRCT) of the chest was performed at baseline as part of routine clinical care. Because of the retrospective design, acquisition parameters were not standardized across all patients. HRCT images were independently reviewed by experienced pulmonologists and classified based on the usual interstitial pneumonia (UIP) pattern as definite, probable, or indeterminate in accordance with established international guidelines [4,10].

Disease progression within the first year was defined as meeting at least two of the following criteria: (i) worsening symptoms of dyspnea or cough; (ii) decline in PFTs: either a >5% decline in FVC or a >10% decline in DLCO; and/or (iii) radiologic progression on HRCT characterized by increased extent of reticulation, traction bronchiectasis, and/or new or worsening honeycombing, consistent with PPF recommendations.

Radiologic progression was assessed by qualitative side-by-side comparison of baseline and approximately 12-month follow-up HRCT scans. Two experienced pulmonologists reviewed the scans independently and resolved discrepancies by consensus; formal semi-quantitative scoring and inter-observer agreement statistics were not performed. Survival duration was measured from the date of IPF diagnosis to the date of death or last follow-up.

Statistical analysis

Descriptive statistics were used to summarize baseline characteristics. Continuous variables were expressed as means ± standard deviation (SD) or medians (interquartile range (IQR)) as appropriate, and categorical variables as frequencies and percentages. Differences between groups (progression vs. non-progression) were evaluated using Student’s t-test or Mann-Whitney U test for continuous variables and Fisher’s exact test for categorical variables. To identify independent predictors of disease progression within one year, exact logistic regression analysis was employed due to the relatively small sample size and potential violation of the assumptions of standard logistic regression [13]. Odds ratios (ORs) with 95% confidence intervals (CIs) were calculated for each candidate variable. Overall survival was analyzed using the Kaplan-Meier method, and survival curves were compared between groups using the log-rank test. Statistical significance was set at a p-value of <0.05. All analyses were conducted using STATA software version 16.1 (Stata Corp, College Station, Texas, United States). Analyses were performed using complete-case data; no imputation was applied.

## Results

Forty-seven patients were included. Mean age was 73.3 ± 7.4 years; 32 were men, and 15 were women. Most were ever-smokers (63.8%), with a mean smoking history of 25.5 ± 26.7 pack-years. The mean baseline modified Medical Research Council Dyspnea Scale (mMRC) score was 1.4 ± 0.9, and 72% reported cough. Laboratory data showed mean WBC at 7896 ± 2396/μL, monocytes at 496 ± 268/μL, LDH at 247 ± 45 U/L, and KL-6 at 1497 ± 1402 U/mL (Table 1). 

**Table 1:** Baseline clinical characteristics of patients with idiopathic pulmonary fibrosis (IPF) (N = 47) The cohort had a mean age of 73 years, was predominantly male, and included a high proportion of ever-smokers. Most patients reported cough and dyspnea, consistent with the typical IPF presentation. mMRC dyspnea scale: modified Medical Research Council dyspnea scale; KL-6: Krebs von den Lungen-6;

Variable		Value
Age (years), mean±SD		73.3 ± 7.4
Sex, n (%)	Male	32 (68.0%)
	Female	15 (32.0%)
Body mass index (kg/m²), mean±SD		25.3 ± 3.9
Smoking history (pack-years), mean±SD		25.5 ± 26.7
Ever-smoker, n (%)		30 (63.8%)
Cough, n (%)		34 (72.3%)
mMRC dyspnea scale score, mean±SD		1.4 ± 0.9
White blood cells (/μL), mean±SD		7896 ± 2396
Monocytes (/μL), mean±SD		496 ± 268
Lactate dehydrogenase (U/L), mean±SD		247 ± 45
KL-6 (U/mL), mean±SD		1497 ± 1402
Antifibrotic agent, n(%)	Nintedanib	26 (55.0%)
	Pirfenidone	21 (45.0%)
Disease progression within 1 year, n (%)	Yes	29 (61.7%)
	No	18 (38.3%)
Survival time (months), mean±SD		46.5 ± 34.1

 PFT results demonstrated a mean FVC of 1.93 ± 0.54 L, %FVC 67.6 ± 14.9, %PEF 75.0 ± 22.6, %FRC 67.6 ± 38.0, %TLC 72.4 ± 13.6, and %DLCO 63.7 ± 25.8. Mean GAP score was 4.9 ± 1.8 and CPI 58.5 ± 26.0. On HRCT, UIP patterns were definite in 20 patients, probable in 19, and indeterminate in eight (Table 2).

**Table 2:** Physiological parameters and HRCT patterns in patients with IPF (n = 47) Note: Pulmonary function tests demonstrated moderate restrictive impairment with reduced diffusing capacity. HRCT patterns were consistent with UIP morphology in the majority of patients. HRCT: high-resolution computed tomography; FVC: forced vital capacity; PEF: peak expiratory flow; FRC: functional residual capacity; TLC: total lung capacity; DLco: diffusing capacity of the lung for carbon monoxide; GAP: gender–age–physiology index; CPI: composite physiologic index; UIP: usual interstitial pneumonia

Variable	Value, mean± SD
FVC, L	1.93 ± 0.54
%FVC	67.6 ± 14.9
%PEF	75.0 ± 22.6
%FRC	67.6 ± 38.0
%TLC	72.4 ± 13.6
%DLco	63.7 ± 25.8
GAP index	4.9 ± 1.8
CPI	58.5 ± 26.0
HRCT pattern, n	Definite UIP: 20 Probable UIP: 19 Indeterminate: 8

Of the 47 patients, 29 (61.7%) met criteria for disease progression within one year. Among them, 22 demonstrated worsening mMRC scores, 12 experienced a ≥5% decline in %FVC, and 25 showed radiologic progression of fibrosis (Table 3). 

**Table 3:** Details of disease progression among patients with IPF (N = 29) Note: Among patients with disease progression, worsening dyspnea and fibrotic changes on HRCT were more common than significant FVC decline, underscoring the multifactorial nature of IPF deterioration. mMRC: modified Medical Research Council dyspnea scale; FVC: forced vital capacity; HRCT: high-resolution computed tomography; IPF: idiopathic pulmonary fibrosis

Domain	Variable	Patients
Symptoms	Worsening dyspnea (increase in mMRC dyspnea scale score)	22
Physiology	Decline in %FVC > 5% (absolute)	12
Imaging	Radiologic progression of fibrotic features (reticular opacity, traction bronchiectasis, or honeycombing)	25

Exact logistic regression analysis revealed that lower baseline %PEF (OR 0.977, p = 0.097) and %TLC (OR 0.953, p = 0.071) showed trends toward association with progression within one year (Table 4). Patients with preserved %PEF and %TLC tended to have slower functional decline. 

**Table 4:** Exact logistic regression analysis for predictors of disease progression in IPF Note: p < 0.05 was considered statistically significant. Lower baseline %PEF (OR 0.977, p = 0.097) and %TLC (OR 0.953, p = 0.071) showed trends toward association with disease progression within one year, suggesting these indices may serve as potential physiological markers of poor outcome despite antifibrotic therapy. IPF: idiopathic pulmonary fibrosis; mMRC: modified Medical Research Council dyspnea scale; LDH: lactate dehydrogenase; PEF: peak expiratory flow; TLC: total lung capacity; DLCO: diffusing capacity of the lung for carbon monoxide; FVC: forced vital capacity

Variable	Odds Ratio	Suff.	2*Pr(Suff.)	95%CI Lower	95%CI Upper
mMRC score	0.7718	39.0	0.5420	0.3715	1.5661
Monocyte (μL)	1.0016	15553.0	0.1889	0.9993	1.0043
LDH (U/L)	1.0057	7043.0	0.4412	0.9920	1.0209
%PEF (L/min)	0.9772	2048.8	0.0966	0.9487	1.0040
%TLC (%)	0.9533	1588.0	0.0710	0.8975	1.0037
%DLCO (%)	0.9889	966.7	0.4571	0.9558	1.0178
FVC ( L)	1.0219	55.9	0.9718	0.3410	3.0675
%FVC (%)	0.9767	1903.6	0.2626	0.9352	1.0170

**Figure 1:**
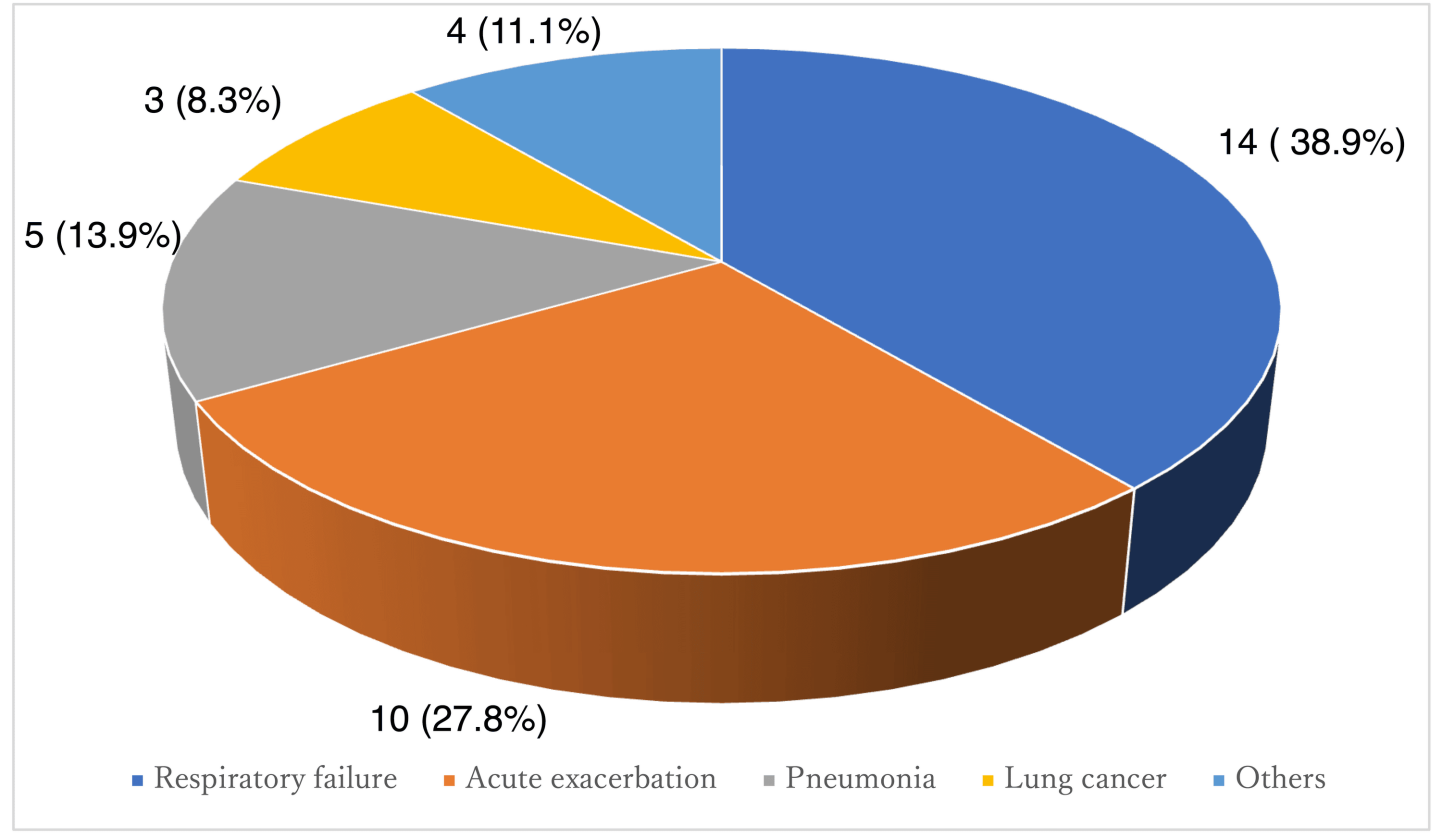
Cause of death (N=36)

**Figure 2:**
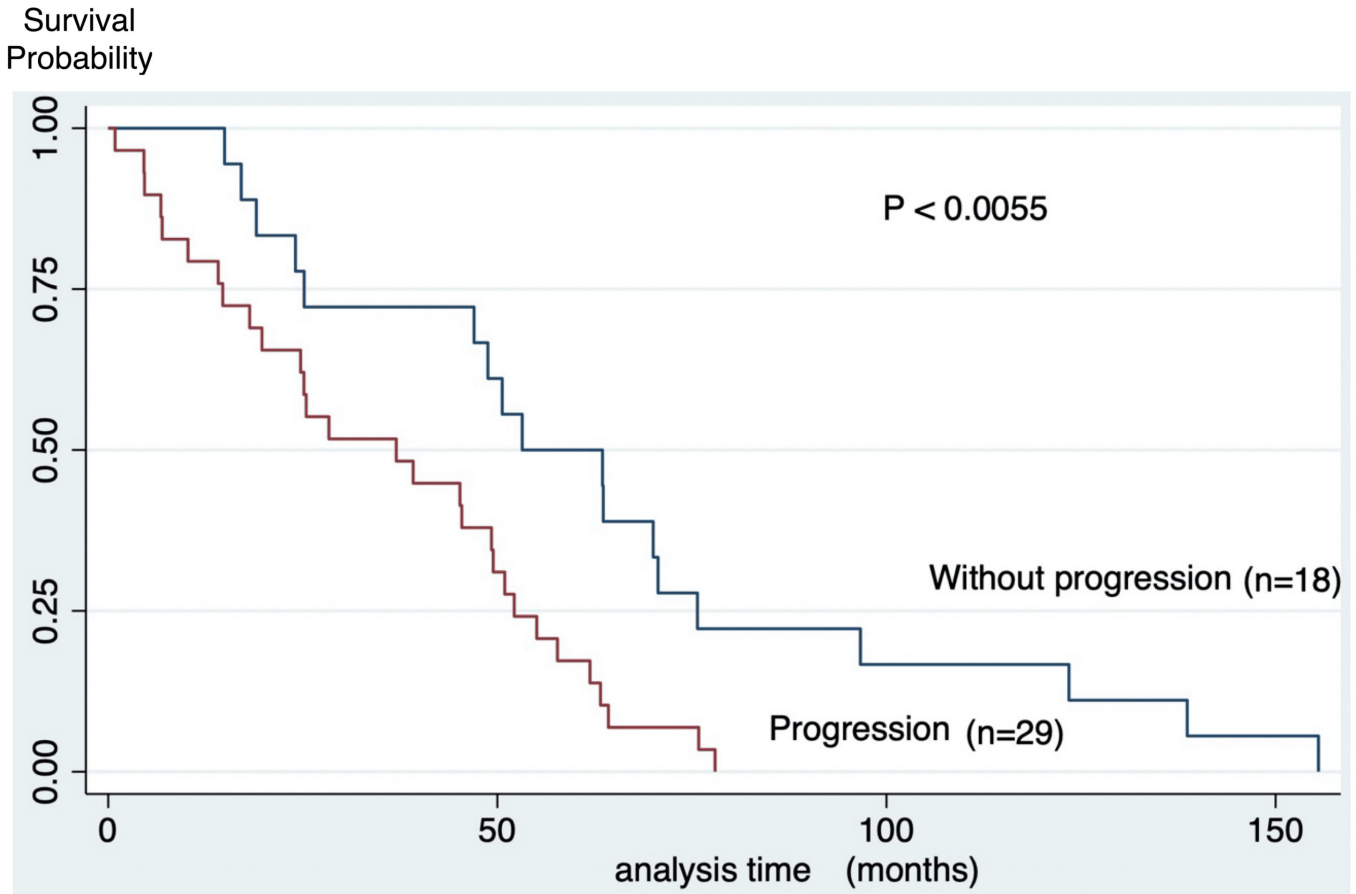
Survival curves according to IPF progression (N=47) Kaplan–Meier curves show significantly reduced overall survival in patients with disease progression within one year compared with those without progression. Abbreviations: IPF, idiopathic pulmonary fibrosis. Note: Survival differences were assessed using the log-rank test; p < 0.05 was considered statistically significant.

During a median follow-up of 24 months, the median survival for the cohort was 46.5 ± 34.1 months. During the observation period, 77% of IPF patients who received antifibrotics died. Among IPF patients who died during the observation period, approximately two-thirds succumbed to respiratory causes, including progressive respiratory failure and acute exacerbations, highlighting the clinical significance of fibrotic progression despite antifibrotic therapy (Figure 1). Kaplan-Meier analysis showed that patients with progression had significantly worse prognosis than those without progression (35.5 vs. 64.3 months) (Figure 2).

## Discussion

This study investigated early disease progression in patients with IPF receiving antifibrotic therapy. We found that lower baseline values of %PEF and %TLC were associated with disease progression within one year, defined using a composite endpoint incorporating symptom worsening, physiological decline, and radiological progression. These findings suggest that even within the context of standard antifibrotic treatment, certain physiological parameters may help identify patients at higher risk for unfavorable clinical trajectories [17-21].

Physiological interpretation of %PEF and %TLC

%PEF, a measure of maximum flow generated during forced expiration, has traditionally been used to assess large airway patency in diseases such as asthma and COPD [22,23]. However, it is increasingly recognized that flow-related parameters such as PEF can also reflect underlying mechanical restriction and parenchymal stiffness in fibrotic lung diseases. In IPF, where fibrosis predominates in the subpleural and lower lung regions, peripheral airway narrowing and traction-induced distortion of the airways can contribute to reduced expiratory flow [24]. Furthermore, TLC is a direct surrogate of total lung volume and may more sensitively reflect the extent of fibrotic remodeling than FVC alone [25].

While FVC and DLCO are standard markers in IPF trials, their sensitivity to early or regional fibrotic change can be limited. DLCO is susceptible to fluctuations based on patient effort and comorbidities such as anemia or pulmonary hypertension. In contrast, TLC decline may reflect architectural distortion and volume loss, and PEF may index the combined effects of lung recoil and airway resistance. Our findings highlight the potential complementary role of these parameters.

Comparison with existing literature

Previous reports have examined PFT-based predictors of IPF outcomes. The ASCEND and INPULSIS trials demonstrated that antifibrotic therapies reduce the rate of FVC decline and delay time to acute exacerbation or death [2,26]. However, even within trial populations, subgroups exhibit heterogeneous progression patterns. Studies by Wells et al. [26,27] and Ley et al. [5] proposed multidimensional indices, CPI and GAP, to better stratify risk. Comprehensive physiologic assessment using indices such as CPI, GAP, and ILD-GAP helps stratify ILD risk [5,13,27], and PFTs remain central to monitoring [14]. These rely on FVC, DLCO, and age or imaging findings but do not include %PEF or %TLC. Our data suggest that %PEF and %TLC may add complementary information beyond %FVC and %DLCO.

Several natural history cohorts have also evaluated lung-volume measures. Baseline TLC (or longitudinal TLC decline) has been associated with disease severity and survival in some cohorts, although it has been less emphasized than FVC because TLC requires plethysmography and is not always measured serially [24,28,29]. In addition, elevated RV/TLC (air trapping) has been described in a subset of patients, particularly when emphysema or small-airway involvement coexists, and has been explored in relation to IPF outcomes, with mixed findings regarding independent prognostic value [30,31]. Taken together, these studies support the concept that lung-volume-based indices may capture physiologic heterogeneity not fully reflected by FVC alone.

To our knowledge, few studies have explored PEF or TLC as prognostic markers in treated IPF cohorts. Our analysis suggests these metrics may provide additional physiologic insight, potentially enhancing risk stratification beyond existing indices. Furthermore, recent recognition of PPF by the 2022 ATS/ERS/JRS/ALAT guideline highlights the need for dynamic monitoring tools applicable across ILD subtypes, including IPF [6]. Our study contributes to this evolving discussion by proposing that certain nontraditional metrics may have practical utility.

Prior studies have also explored lung volume metrics in IPF cohorts. For example, real-world registry data have evaluated baseline TLC% alongside established predictors and suggested that TLC% may carry prognostic information, although its contribution may be attenuated when functional capacity measures (e.g., six-minute walk distance (6MWD)) are included in multivariable models [23]. In addition, more recent analyses have examined air trapping quantified by RV/TLC in IPF; while RV/TLC was associated with clinical features and outcomes in univariable analyses, it was not consistently retained as an independent prognostic factor after adjustment, emphasizing the need to clarify when lung volume/flow metrics add incremental value beyond conventional indices [25]. Our findings extend this literature by focusing on antifibrotic-treated IPF and identifying baseline %TLC and %PEF as candidate parameters associated with early progression, supporting further study in larger prospective cohorts.

Radiologic-physiologic correlation and limitations

Our composite definition of disease progression incorporated radiologic progression, including worsening of reticulation, traction bronchiectasis, or honeycombing. While HRCT is the gold standard for diagnosis and staging in IPF, its use in longitudinal monitoring is debated due to radiation exposure and subjective interpretation variability. Nonetheless, radiologic changes-particularly increases in fibrotic burden-have been shown to correlate with mortality and physiologic decline [28-32]. Our study supports this relationship and reinforces the role of imaging in defining progression.

Contemporary diagnostic frameworks from ATS/ERS/JRS/ALAT and the Fleischner Society have standardized HRCT patterns and strengthened clinico-radiologic integration [4,33]. Our composite progression definition, aligned with PPF guidance [6,17,18], captured radiologic worsening even when %FVC changes were modest. However, challenges remain in quantifying radiologic progression objectively. Semiquantitative or automated image analysis software may improve reproducibility in future studies [31]. Additionally, our cohort had a relatively high rate of progression (61.7%), possibly due to the inclusion of radiologic criteria, which may detect changes not yet reflected in physiology.

Timing of antifibrotic initiation and clinical implications

It is now widely accepted that earlier initiation of antifibrotic therapy is associated with better outcomes. Retrospective data from real-world cohorts have shown that treatment delay is linked to worse survival, particularly in patients with preserved lung function [16]. Our findings align with this paradigm by suggesting that patients with reduced %PEF or %TLC may already be on a steeper decline trajectory despite therapy. Heterogeneity of response despite antifibrotics is well documented in trials and real-world cohorts [2,3,19,21,26].

Expert opinion and observational data advocate early initiation of antifibrotics to optimize outcomes [17,19,20]. Our finding that reduced %PEF and %TLC are linked to progression supports the notion that subtle physiologic impairment may signal a narrowing therapeutic window [23,26,27]. This may support the concept of “therapeutic window”-an optimal phase in the disease course when intervention yields the greatest benefit [32-35]. Clinicians may therefore consider being attentive to subtle physiological deficits, even if traditional markers such as FVC remain relatively preserved. %PEF and %TLC may serve as early warning signs prompting consideration of closer monitoring or trial enrollment.

Clinical relevance and future directions

From a practical standpoint, %PEF and %TLC are obtainable via standard PFTs, require no specialized equipment, and can be tracked longitudinally. Integrating these into routine clinical evaluation may provide a more comprehensive picture of disease behavior. Furthermore, incorporating these variables into future clinical trials may enhance endpoint sensitivity, particularly in early-stage or slowly progressing patients. The broader construct of PPF emphasizes dynamic monitoring across ILD subtypes, not solely IPF [6,17,18,27].

Quantitative CT approaches may further refine longitudinal assessment and prediction [36-40]. Future studies could combine %PEF and %TLC with quantitative imaging and biomarkers such as KL-6 to build multiparametric risk models. Prospective studies are needed to validate these findings in larger cohorts and to explore the mechanistic basis for the association between flow-volume metrics and fibrotic remodeling. Additional variables such as oscillometry, lung compliance, or quantitative imaging may also complement our approach.

Limitations of the study

This study has several limitations. First, this was a retrospective single-center study with a modest sample size, which limits generalizability and statistical power. Second, although all patients initiated antifibrotic therapy, detailed information on treatment adherence, dose reductions, temporary interruptions, and permanent discontinuation was not analyzed; variations in treatment exposure may have influenced outcomes. Third, disease progression was defined using a composite endpoint that included symptomatic, physiologic, and radiologic criteria; radiologic worsening was assessed qualitatively on side-by-side HRCT comparison without semi-quantitative scoring or formal inter-observer agreement metrics. Finally, although we adjusted for key clinical variables, residual confounding (e.g., comorbidities, frailty, and unmeasured biomarkers) cannot be excluded.

Larger prospective multicenter studies are required to validate these findings and clarify the role of %PEF and %TLC in risk stratification. In particular, the small sample size (n=47) and limited number of progression events (n=29) reduced statistical power, and the observed associations did not meet conventional thresholds for statistical significance. Accordingly, multivariable adjustment was not feasible, and residual confounding cannot be excluded. HRCT acquisition protocols were not standardized, and radiologic progression was assessed qualitatively without formal inter-observer reliability metrics; some misclassification is possible. In addition, antifibrotic treatment exposure was assessed based on initiation, but detailed treatment-course variables-such as adherence, dose intensity, dose reductions, temporary interruptions, and permanent discontinuation-were not systematically captured and therefore could not be incorporated into outcome analyses. These unmeasured treatment factors may have influenced progression and survival and should be addressed in prospective studies.

## Conclusions

In this retrospective study of IPF patients treated with antifibrotics, lower baseline %PEF and %TLC showed trends toward association with early disease progression within one year. Given the retrospective design and limited power, these findings should be considered hypothesis-generating.

These findings suggest that routinely available pulmonary function indices beyond %FVC and %DLco may serve as potential adjunctive markers to identify patients at higher risk of early progression. Prospective, multicenter studies are needed to validate these associations and determine whether incorporating %PEF and %TLC improves prognostic or treatment-response models. They are not sufficient to guide standalone clinical decision-making without prospective multicenter validation.
